# Mortality surveillance during the COVID-19 pandemic

**DOI:** 10.2471/BLT.20.263194

**Published:** 2020-06-01

**Authors:** Philip Setel, Carla AbouZahr, Emily B Atuheire, Martin Bratschi, Emily Cercone, Oliver Chinganya, Benjamin Clapham, Samuel J Clark, Carlie Congdon, Don de Savigny, Adam Karpati, Erin Nichols, Robert Jakob, James Mwanza, William Muhwava, Petra Nahmias, Elizabeth M Ortiz, Akhona Tshangela

**Affiliations:** a Vital Strategies Bloomberg Philanthropies Data for Health Initiative, 100 Broadway, 4th Floor, New York, NY 10006 United States of America (USA).; b Mortality Surveillance Programme, Africa Centres for Disease Control and Prevention, Addis Ababa, Ethiopia.; c US Centers for Disease Control and Prevention, National Center for Health Statistics, Hyattsville, USA.; d African Centre for Statistics, United Nations Economic and Social Commission for Africa (UNECA), Addis Ababa, Ethiopia.; e Department of Sociology, The Ohio State University, Ohio, USA.; f Swiss Tropical and Public Health Institute, University of Basel, Basel, Switzerland.; g Division for Data and Analysis, World Health Organization, Geneva, Switzerland.; h Statistics Division, United Nations Economic and Social Commission for Asia and the Pacific, Bangkok, Thailand.

During an epidemic, rapid mortality surveillance provides essential information to formulate an evidence-based response. Weekly counts of deaths are a key indicator of overall epidemic impact and trajectory.^1^^,^^2^ Enumeration of all deaths, when compared to historically expected mortality, produces a picture of excess death, capturing both the direct burden of the epidemic and its indirect mortality burden, caused by disruptions to the access, use and provision of health-care services. Such actionable public health intelligence can overcome the ambiguities of just measuring cases and deaths linked to the infectious disease causing the epidemic. Measuring excess death would therefore be useful in the countries’ response to the coronavirus disease 2019 (COVID-19) pandemic.

Rapid mortality surveillance comprises both facility- and community-based components and depends on both data availability and transmissibility. Networks of community and health facility sites reporting deaths by age, sex and location on a daily or weekly basis provide an essential input to the outbreak response, including tracking the epidemic trajectory after adjusting public health and social measures.^3^ This type of rapid surveillance has been implemented in numerous settings, including in Europe and Africa, and its feasibility has been demonstrated even under difficult conditions, such as mass displacement.^4^^–^^9^

Facility-based surveillance may be implemented by medical records staff, observing proper infection control measures to protect themselves, including avoiding patient-care areas; observing workplace physical distancing measures; rigorously practicing hand hygiene; and use of personal protective equipment. A simple list of deaths by age, sex and usual residence transmitted weekly from a selected set of sentinel facilities is the starting point. One approach to rapid mortality surveillance is to leverage existing routine health information systems, COVID-19-specific rapid data collection platforms or existing surveillance platforms, such as integrated disease surveillance and response.

Community-based surveillance may be critical where high numbers of deaths occur outside of health facilities – a situation that may worsen should health systems become overwhelmed. Community surveillance can be done by community-based health-care providers or other frontline workers whose existing remit includes the notification of vital events, provided that infection prevention measures can be observed to protect those collecting data.

Ideally, with little lag-time, the system could generate weekly counts of deaths by demographic group and location. Real-time data can be compared to predicted deaths from historical data. For populations without historical data, the initial period of complete reporting can provide a baseline against which to compare emerging trends.

Rapid mortality surveillance may provide policy-makers with up-to-date status reports, including spread into new areas or previously unaffected populations. This surveillance can also help target, prioritize and monitor the effectiveness of prevention and response strategies. Countries need real-time awareness of the distribution and magnitude of all direct and indirect health impacts of the COVID-19 pandemic. Establishing, scaling and improving upon rapid mortality surveillance would contribute to fulfilling this need, as well as preparing for future outbreaks.
